# Turin International Exhibition 1911. The British Section

**Published:** 1911-02-18

**Authors:** Boverton Redwood

**Affiliations:** Chairman Chemical Industries Committee. Board of Trade (Exhibitions Branch)


### TURIN INTERNATIONAL EXHIBITION 1911.
### THE BRITISH SECTION.

Sir,—It has been decided that in the British Section of the approaching Turin Exhibition, chemical and physical apparatus shall be shown as far as possible in a truly practical and novel manner; and I trust you will afford me through the medium of your valuable columns an opportunity of giving information to the public regarding the scheme.

Generally speaking, no means are provided at exhibitions for demonstrating the utility of the instruments exhibited ; and it has been felt that it would be a very great improvement to show apparatus as it would be used in a laboratory. Accordingly, for the British Chemical Section of the Turin Exhibition arrangements are now approaching completion by which, it is anticipated, there will be on view at least two well-equipped chemical laboratories, with such work going on as will effectively illustrate various interesting processes. In addition, there will be a large space available for the display in showcases of chemical products and apparatus not in use in the laboratories. Smaller rooms will be provided for certain special appliances. The Court devoted to Scientific Instruments will be of similar design. Here also arrangements are being made for the display of apparatus ready for work, electric supply, where needed, being provided. The equipment of a large dark-room is under consideration, and in this projection-apparatus, such as oscillographs, spectroscopes, optical lanterns, and photometers could be shown to advantage. The organisation of the exhibits referred to has been placed by the Exhibitions Branch of the Board of Trade in the hands of Dr. F. Mollwo Perkin, under the direction of a joint Sub-Committee of the Chemical Industries Committee and the Mathematical and Scientific Instruments Sub-Committee. This joint Sub-Committee considers that exhibitors could not have more favourable conditions for demonstrating the merits of their exhibits than those which this new arrangement will afford, and that at the same time in the way of instruction by such demonstrations the visiting public will be greatly benefited.—Your obedient servant,

Boverton Redwood,
Chairman Chemical Industries Committee.

Board of Trade
(Exhibitions Branch), Feb. 2.

